# Measurement of cell compressibility changes during epithelial–mesenchymal transition based on acoustofluidic microdevice

**DOI:** 10.1063/5.0072126

**Published:** 2021-11-08

**Authors:** Qibin Fu, Yan Zhang, Tuchen Huang, Ying Liang, Yang Liu

**Affiliations:** 1 Sino-French Institute of Nuclear Engineering and Technology, Sun Yat-sen University, Zhuhai 519082, China; 2 Department of Radiation Oncology, Shenzhen Center, Cancer Hospital Chinese Academy of Medical Sciences, Shenzhen 518116, China

## Abstract

Epithelial–mesenchymal transition (EMT) confers migratory and invasiveness abilities on cancer cells, as well as leading to changes in biomechanical properties and cytoskeletal structure. Cell mechanical properties are considered to be promising label-free markers for diagnosis of cancer metastasis. In this work, cell compressibility, a novel and important parameter of cell mechanical properties, was measured directly and quickly using a specially designed acoustofluidic microdevice. The compressibilities of cells with different metastatic potentials were investigated. Based on a comparison of the measurement results, non-metastatic cells exhibited lower compressibility than metastatic cells. The correlation between cell compressibility and EMT status was further studied; the results showed that the acquisition of mesenchymal status was accompanied by an increase in cell compressibility. These findings imply strong correlations among cell compressibility, EMT status, and invasiveness. Therefore, cell compressibility represents a novel biomechanical marker for evaluating malignant transformation and metastasis of cancer.

## I. INTRODUCTION

In the process of cancer metastasis, cancer cells with invasive ability shed from the primary tumor, migrate, invade *in situ*, infiltrate the circulatory system, then extravasate from blood vessels and form metastases at the distal site.^1^ During this process, the mechanical properties of cancer cells change significantly, and mechanotransduction of these properties into biochemical signals is accomplished through various signaling pathways.^1^ Studies in recent years have found that cell mechanical properties could serve as new biomarkers for the diagnosis of malignant development of cancer. In several cell types, cancer cells have been found to be less stiff than non-cancerous cells.^2,3^ Moreover, metastatic tissue was less stiff than primary tumor tissue.^4^ The stiffness and deformability of cancer cells are restrictive factors affecting migration.^5^ Decreasing cell stiffness by pharmacologic inhibition could increase invasiveness.^6^ Collectively, these results indicate a correlation between cellular mechanical properties and cancer metastasis.

As described above, in the initial and key step of cancer metastasis, cancer cells deform and invade *in situ*, thereby leaving the primary tumor. Epithelial–mesenchymal transition (EMT) can endow cancer cells with enhanced migratory ability and invasiveness.^7–9^ The EMT process mainly manifests as the downregulation of epithelial markers (E-cadherin, cytokeratin-18, occludins, etc.) and the acquisition of mesenchymal markers (vimentin and α-SMA, etc.).^7–9^ The transcription factors that induce EMT not only regulate cell polarization, cytoskeletal structure, and degradation of extracellular matrix but also inhibit key genes of the epithelial phenotype.^10,11^ Therefore, EMT may have a critical role in regulating cellular mechanical signals.

There are several conventional methods to measure cellular mechanics. Atomic force microscopy has been widely used to measure cell stiffness (Young's modulus).^3,12^ Cell viscoelasticity can be obtained by deforming cells using various methods, such as suction pressure induced by micropipete aspiration,^13^ magnetic beads based on magnetic tweezers,^14^ or a focused laser applied by optical tweezers.^6^ Microfluidic constriction channels can also be used to study mechanical properties during cell migration.^15^ However, these methods cause cell damage due to contact or local deformation, making subsequent live cell experiments impossible. In addition, the experimental throughput of these methods is limited and the experiment preparation is relatively complicated. In the present work, a fast and direct measurement of cell compressibility was implemented on a microfluidic system based on acoustic radiation technology.^16^ As the acoustic radiation force is a non-contact force, it can avoid problems such as inaccurate measurement and cell damage caused by contact or local deformation. Moreover, the cells are in suspension, so the measurements can be performed consecutively using a continuous flow platform combined with an acoustic standing wave. The measurement throughput can be controlled by adjusting the cell concentration. Given these advantages, this approach represents a promising label-free method for measuring cellular mechanical properties.

Previous work showed that cancer cells had higher cell compressibility in comparison with normal cells.^16^ Recent studies showed that cells with poor metastatic potential had lower compressibility in head and neck cancer^17^ and breast cancer.^18^ These results indicated that there may be a link between cell compressibility and cellular invasiveness or metastasis. As EMT can induce the migration and invasion of cancer cells,^19^ it is important to study the evolution of cell compressibility during the EMT process. However, the changes in cell compressibility after EMT are still unknown.

In this work, the compressibilities of cancer cells with different invasiveness were measured by an acoustofluidic microdevice. Furthermore, EMT was induced by growth factors in these cells, and the change in cell compressibility after EMT induction was studied. The acoustofluidic method was successfully applied to measure the compressibilities of cells with different EMT statuses, and the correlation between EMT status and cell compressibility was determined. According to our results, cell compressibility could be used as a novel indicator to monitor the EMT process, with potential applications in cancer diagnosis.

## II. MATERIALS AND METHODS

### A. Cell cultivation

Human breast carcinoma cell lines MCF7 and MDA-MB-231 and the human lung adenocarcinoma cell line A549 were purchased from the American Type Culture Collection. A549 and MCF7 cells were, respectively, cultured in Dulbecco's modified Eagle medium (DMEM) with 4.5 g/l glucose or minimum essential medium (MEM) supplemented with 10% fetal bovine serum (FBS). For MDA-MB-231 cells, the complete medium was L15 medium containing 10% FBS, and the cells were incubated in a flask with a plug seal cap. All cell lines were cultivated at 37 °C and 5% CO_2_ concentration.

### B. Experimental device and procedures

**FIG. 1.**
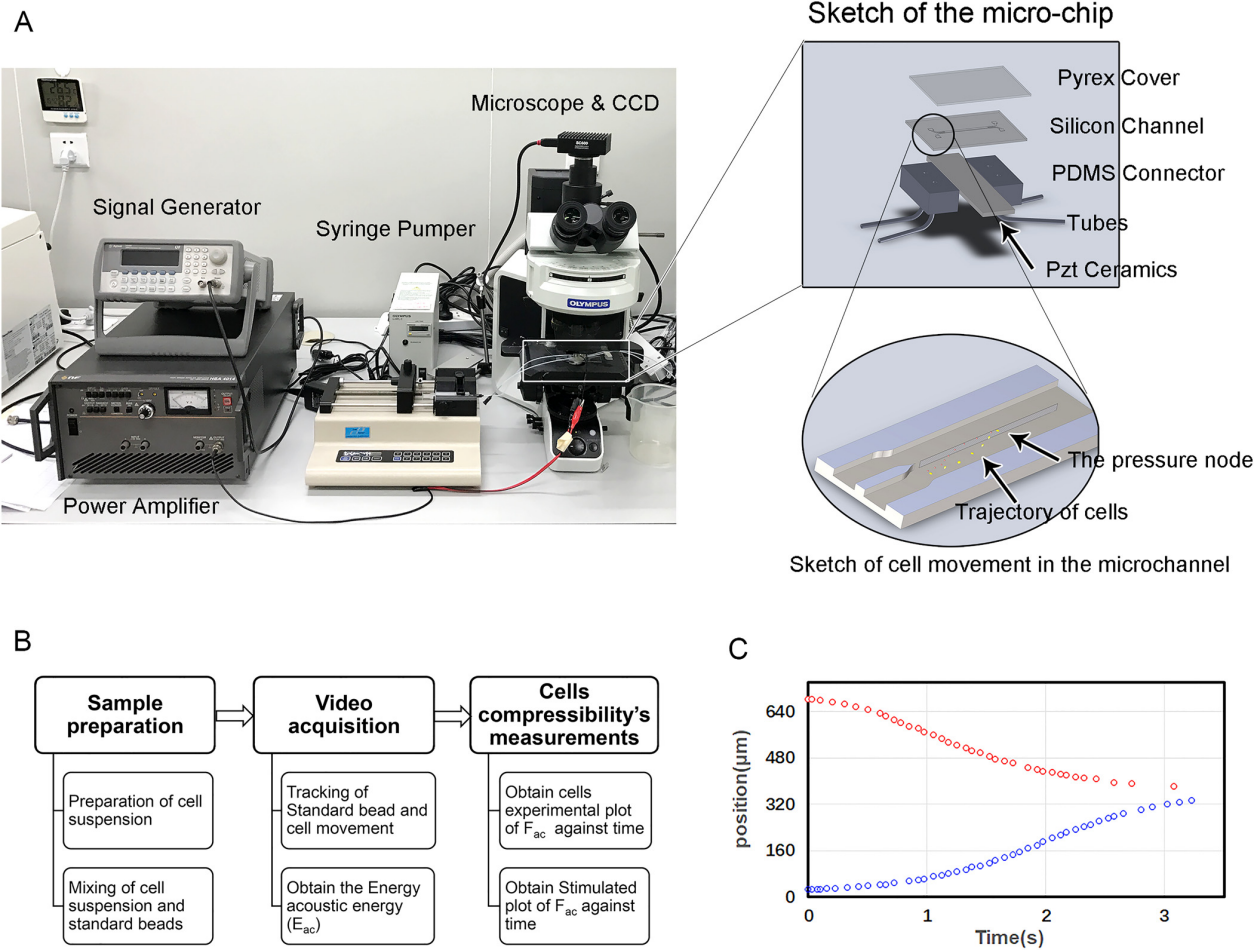
Experimental setup and procedures. (a) Experimental device and schematic diagram of the microfluidic chip. (b) General procedures for measuring cell compressibility. (c) Cells from different locations converged to the center of the microchannel.

The experimental device is shown in Fig. 1(a). A microchannel with a rectangular cross section (740 *μ*m width and 100 *μ*m depth) was fabricated on a silicon wafer by reactive ion etching. The channel was sealed on top with a piece of transparent Pyrex and attached beneath the wafer by a piezo-electric ceramic to provide the acoustic field. The ceramic was selected to work in thickness mode with a first primary resonance frequency of 1 MHz, corresponding to that of the resonant standing wave formed in the channel. The sinusoidal electric signal used to excite this ceramic was provided by a function generator (Agilent 33220A) and subsequently amplified by a power amplifier before being applied to the ceramic. Cells and standard polystyrene beads were suspended in phosphate-buffered saline (PBS) and pumped through the microchannel. Notably, to reduce the deviation of cell compressibility caused by the change of acoustic energy density, standard beads were always introduced with the cells to determine the acoustic energy density. The trajectories of cells and standard polystyrene beads were observed under a microscope (Olympus BX53) and recorded with a CCD camera. The positions of beads or cells at different times were tracked in the recorded videos by our MATLAB codes.

The experimental procedures for this study are shown in Fig. 1(b). First, to reduce the influence of variations in the acoustic field, polystyrene beads with 6 *μ*m diameter were mixed in the cell suspension in each independent experiment. Standard beads served as a reliable control because of their fixed physical properties (size, density, and compressibility). For the preparation of cell suspensions, cells with or without EMT induction were enzymatically digested and resuspended in PBS. The density of cells was approximately 500 cells per microliter. During the passage of each sample through the channel, an acoustic standing wave field was activated to provide a lateral external acoustic radiation force. Under the action of this acoustic force, cells and standard beads converged to the central line of the channel when flowing, which meant they would move laterally across the channel. Different cells or beads have different converging trajectories [Fig. 1(c)]. Second, the movements of cells and standard beads were recorded by a CCD camera, and the corresponding motion trajectories were extracted by image processing. The measured trajectories of standard beads were used to obtain the acoustic energy density [*E* in Eq. (1)] by fitting with the corresponding theoretical trajectories described in previous work.^16^ Finally, the compressibility (*β*) of the cell was calculated by trajectory fitting with the obtained acoustic energy density parameter.

### C. Principle of measuring the compressibility of standard beads and cells

According to the microfluid hypothesis, cells (or beads) that are carried by fluid through a straight microchannel with a rectangular cross section will flow along the straight streamline that is parallel to the channel's sidewall. When a sinusoidal acoustic standing wave is established between the channel's sidewalls, cells flowing through the channel will experience a time-averaged non-contact acoustic force.^20^ The relationship between a cell's compressibility and the acoustic force can be described using a simplified equation [Eq. (1)], when the suspended cell is assumed to be a sphere,

$$F_{ac}=-\frac{4}{3}\pi r^{3}kE\left(\frac{5\rho_{c}-2\rho_{f}}{2\rho_{c}+\rho_{f}}-\frac{\beta_{c}}{\beta_{f}}\right)\sin(2kd),$$ (1)

where *r* is the radius of the suspended objects, *ρ* is the density, *β* is the compressibility, the subscripts *c* and *f* indicate the cell and fluid, respectively, and *d* is the distance from the nearest acoustic pressure node. The acoustic field with standing wave is characterized by two parameters: the wave number *k* and the energy density *E*.

Furthermore, the spherical cell is affected by the hydrodynamic force,

$$\vec{F_{D}}=3\pi \mu D({\vec{v}}_{f}-{\vec{v}}_{c}),$$ (2)

where *μ* is the dynamic viscosity of the fluid, *D* is the diameter of the cell, $\vec{v}$ is the velocity vector, and the subscripts *c* and *f* indicate the cell and fluid, respectively.

Owing to the differences in physical characteristics (including compressibility and size), different cells have different trajectories when passing through the channel. Theoretically, these trajectories can be solved by Newton's second law. Considering that the size of a cell is quite small compared with that of the channel, it can be assumed that cell motion along the channel follows the streamline of background fluid. Along the transverse direction, cells bear the acoustic radiation force and hydrodynamic drag force. Supposing that the acoustic radiation force is established on the cell instantaneously once the cell flows into the acoustic field, the cell will be gradually accelerated from its stationary state in y direction and converge to the pressure node. This acoustic radiation force is a function of the position [shown in Eq. (1)]. Thus, the movement process along the y direction, which is used in the cell compressibility measurement, becomes more complex than the movement along the x direction. Therefore, in order to improve the measurement accuracy of cell compressibility, the transverse motion (y direction) is described as follows:

$$m\frac{d^{2}y}{d^{2}t}=F_{ac}(y,t)+F_{D}(y,t),$$ (3)

where *F_ac_* and *F_D_* are described by Eqs. (1) and (2), respectively.

Using the fourth-order Runge–Kutta method, the numerical solution of the cell trajectory under the acoustic field was obtained.

### D. EMT induction

To induce EMT in MCF7 cells, starvation pretreatment was performed in MEM containing 1% FBS for 24 h. Then, MCF7 cells were grown in their original complete medium supplemented with growth factors [20 ng/ml fibroblast growth factor-basic (bFGF) and 20 ng/ml epidermal growth factor (EGF)] for 3 days (referred to subsequently as iEMT 3d) or 5 days (iEMT 5d). To induce EMT in A549 cells, cells were grown in their original complete medium containing 5 ng/ml TGFβ for 48 h (iEMT 2d) or 72 h (iEMT 3d). Cell morphology was captured with a phase-contrast microscope (Thermo Scientific, EVOS) with a 10× objective lens.

### E. Immunofluorescence

Cells were adhered to a coverglass. After EMT induction, cells were fixed with 4% paraformaldehyde (P0099, Beyotime Biotechnology), before being permeabilized and blocked with 0.3% Triton X-100 and 5% bovine serum albumin in PBS. Cells were then incubated with primary antibodies overnight at 4 °C. To characterize EMT status, cells were labeled with E-cadherin antibody (Cell Signaling Technology, 14472S) and vimentin antibody (Cell Signaling Technology, 5741T). FITC-Goat Anti-Mouse IgG (Proteintech) and Texas Red-Goat Anti-Rabbit IgG (Proteintech) were used as secondary antibodies. Cell nuclei were stained with DAPI. Images were obtained using a fluorescence microscope (Olympus BX53) with a 40× or 100× oil objective lens.

### F. Invasion assay

Cell invasion was assessed using Corning Biocoat cell culture inserts (8 *μ*m pore size) covered with a thin layer of Matrigel according to the manufacturer's instructions. The upper chamber was inoculated with 5 × 10^4^ cells in a medium without FBS. The lower chamber was filled with a medium containing 10% FBS as a chemical attractant. After overnight cultivation at 37 °C, the residual cells on the upper surface of the membrane were removed with a cotton swab, and invasive cells attached to the lower side of the membrane were stained with crystal violet. The stained cells in five different random fields were counted under a microscope at a magnification of 200×.

### G. Migration assay

A549 cells with or without EMT induction were grown to near-confluence in 24-well plates. Then, a scratch was made on the monolayer using a scratcher (SPL Scar). The monolayer was rinsed three times with PBS and placed in the original complete medium. After 24 h incubation, the scratch was imaged by a phase-contrast microscope (Thermo Scientific, EVOS) with a 10× objective lens. The area of cell migration was measured using ImageJ software.

### H. Western blotting

Whole-cell protein lysates of A549 cells or EMT-induced A549 cells were prepared in RIPA lysis buffer containing protease inhibitors. Pierce BCA (Thermo Scientific) assay was used to measure the protein concentrations of cell lysates. Protein samples (20 *μ*g for each) were separated by 10% sodium dodecyl sulfate polyacrylamide gel electrophoresis and then transferred to polyvinylidene membranes. The membranes were then blocked with 5% skimmed milk in Tris-buffered saline with Tween 20 (TBST) solution. The blots were incubated with the same anti-E-cadherin and anti-vimentin antibodies as described above in blocking solution at 4 °C overnight. After rinsing with TBST buffer, the blots were incubated with secondary antibodies of the corresponding species conjugated with horseradish peroxidase (Beyotime Biotechnology). After incubation for 1 h, the blots were rinsed with TBST buffer and developed with an enhanced chemiluminescence detection system (Tanon).

### I. Statistical analysis

For each experiment in this study, at least three independent replicates were performed. The results are presented as mean ± SD. Student's *t*-test and chi-square analysis were used to assess significance, which was defined as *P* < 0.05 (significant difference) or *P* < 0.01 (extremely significant difference). Statistical software SPSS was used for correlation analysis. The relationships of cell compressibility with cell invasiveness, cell migration, and the expression of vimentin were all evaluated by Pearson's product–moment correlation and expressed as correlation coefficients (R).

## III. RESULTS AND DISCUSSION

### A. Measurements of the compressibility of breast cancer cells with different metastatic potentials

**FIG. 2.**
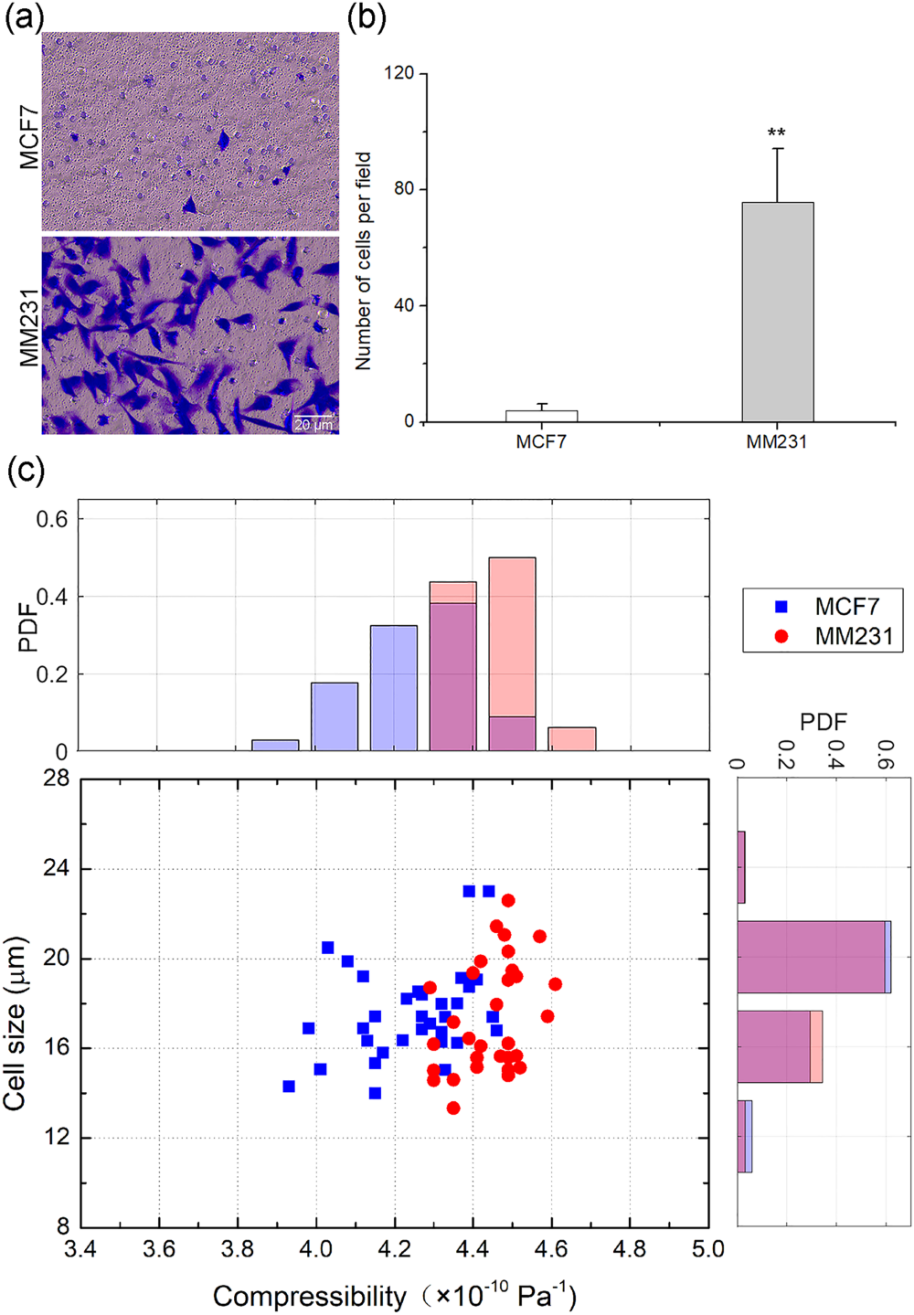
Invasiveness, cell compressibility, and cell size in different types of metastatic breast cancer cells. (a) Images of invasive MCF7 and MM231 cells on the underside of the membrane. Scale bar = 20 *μ*m. (b) Numbers of invasive cells per field under 20× objective lens. MM231 cells showed stronger invasion ability than MCF7 cells. **P < 0.01. (c) Scatter plots and probability distribution plots of cell size and cell compressibility. Each dot represents the corresponding information of one MCF7 cell (blue dot) or one MM231 cell (red dot). As shown in the upper panel, MM231 cells (red column) had higher cell compressibility than MCF7 cells (blue column). As shown in the right panel, MCF7 cells and MM231 cells had similar size distributions.

The designed acoustofluidic microdevice was used to measure the cell compressibility of breast cancer cells with different metastatic potential. Non-metastatic MCF7 cells and their metastatic counterpart MDA-MB-231 cells (referred to subsequently as MM231 cells) were selected for comparison. The invasiveness and metastasis of cancer cells were assessed *in vitro* using the classical invasion experiment.^21^ The invasion chamber with Matrigel matrix mimicked the basement membrane, occluded the membrane pores and prevented non-metastatic MCF7 cells from penetrating into the membrane and migrating. By contrast, metastatic MM231 cells secreted matrix metalloproteinase,^22^ which enzymatically degraded the Matrigel matrix and then deformed and were able to pass through the membrane pores [Fig. 2(a)]. As a result, the metastatic MM231 cells exhibited a significantly higher invasive ability than the non-metastatic MCF7 cells [Fig. 2(b), *P* < 0.01].

When subjected to the acoustic radiation force, cells move toward the pressure node at the center of the channel [Fig. 1(c)]. The compressibility of cells is obtained by fitting the theoretical and experimental trajectories of standard beads and cells. As shown in Fig. 2(c), the two breast cancer cell lines showed very similar distributions of cell size (the cell diameters were 17.6 ± 2.1 *μ*m for MCF7 and 17.3 ± 2.5 *μ*m for MM231). However, the compressibility of the invasive MM231 cells was obviously higher than that of the non-invasive MCF7 cells [4.44 ± 0.09 × 10^−10^ Pa^−1^ for MM231 vs 4.24 ± 0.14 × 10^−10^ Pa^−1^ for MCF7, *P* < 0.01, Fig. 2(c)]. According to Pearson's correlation analysis, the correlation between cell compressibility and invasiveness was very high (correlation coefficient R = 0.964, *P* < 0.01). These results indicated that the invasive cancer cells were more compressible than the non-invasive cancer cells. Our results were consistent with those of other studies that showed that the stiffness of cancer cells derived from a patient or cell line was negatively correlated with migration, invasion, and metastatic spreading.^4,6,13^ Therefore, the results clearly indicate that changes in compressibility may be a sign of cancer aggressiveness.

**FIG. 3.**
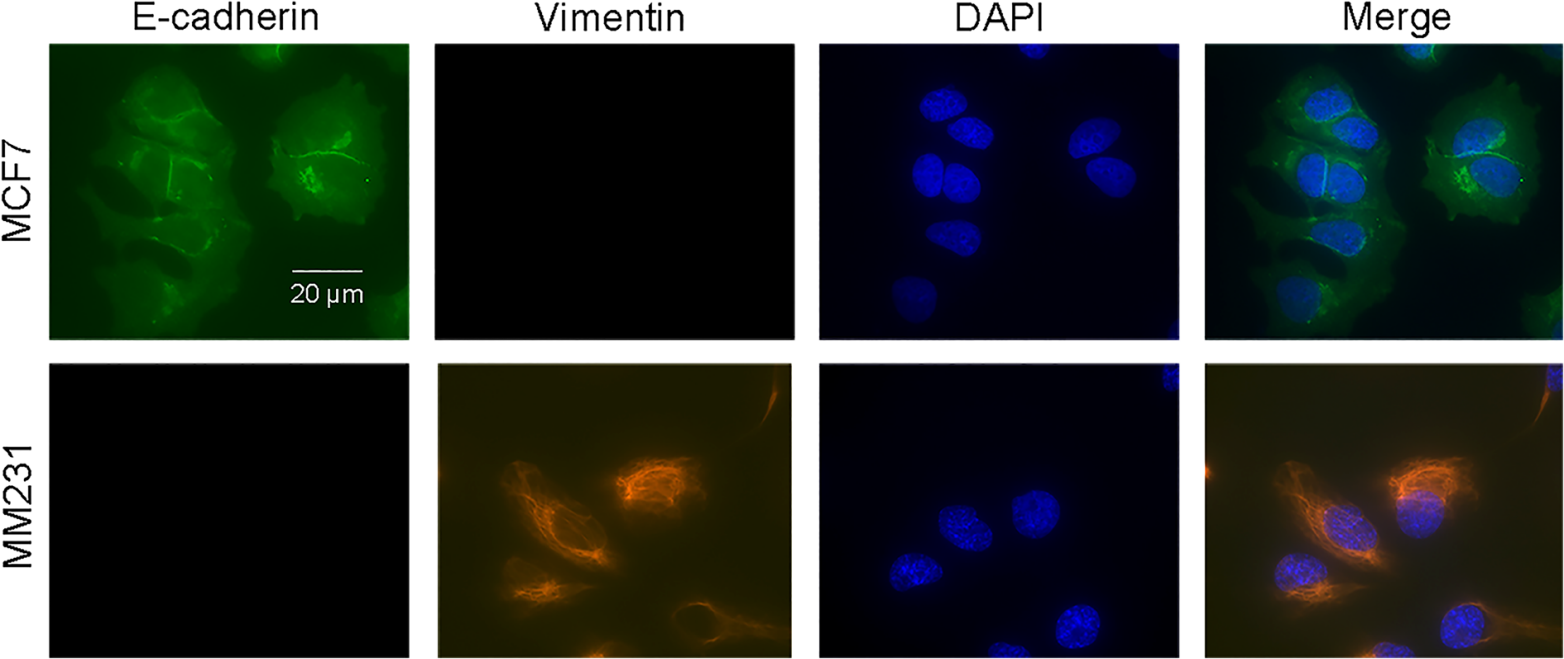
Fluorescence images showing the expression of EMT-related biomarkers in different types of metastatic breast cancer cells. Non-metastatic MCF7 cells expressed E-cadherin protein (epithelial marker, green), while metastatic MM231 cells expressed vimentin protein (mesenchymal marker, red). DAPI was used to stain cell nuclei (blue). The merged image is a superposition of all images. Cells were visualized with a 100× oil objective lens. Scale bar = 20 *μ*m.

Notably, non-invasive MCF7 cells with lower compressibility expressed E-cadherin (a marker of EMT downregulation) but not vimentin (a marker of EMT upregulation). By contrast, the highly invasive MM231 cells with higher compressibility only expressed vimentin (Fig. 3). The gene expression patterns of highly metastatic cells also exhibit the characteristics of the mesenchymal state.^23^ Taken together, these results indicate that it is of great significance to study the relationship between cell compressibility and EMT status.

### B. Correlation between cell compressibility and molecular signature of EMT

**FIG. 4.**
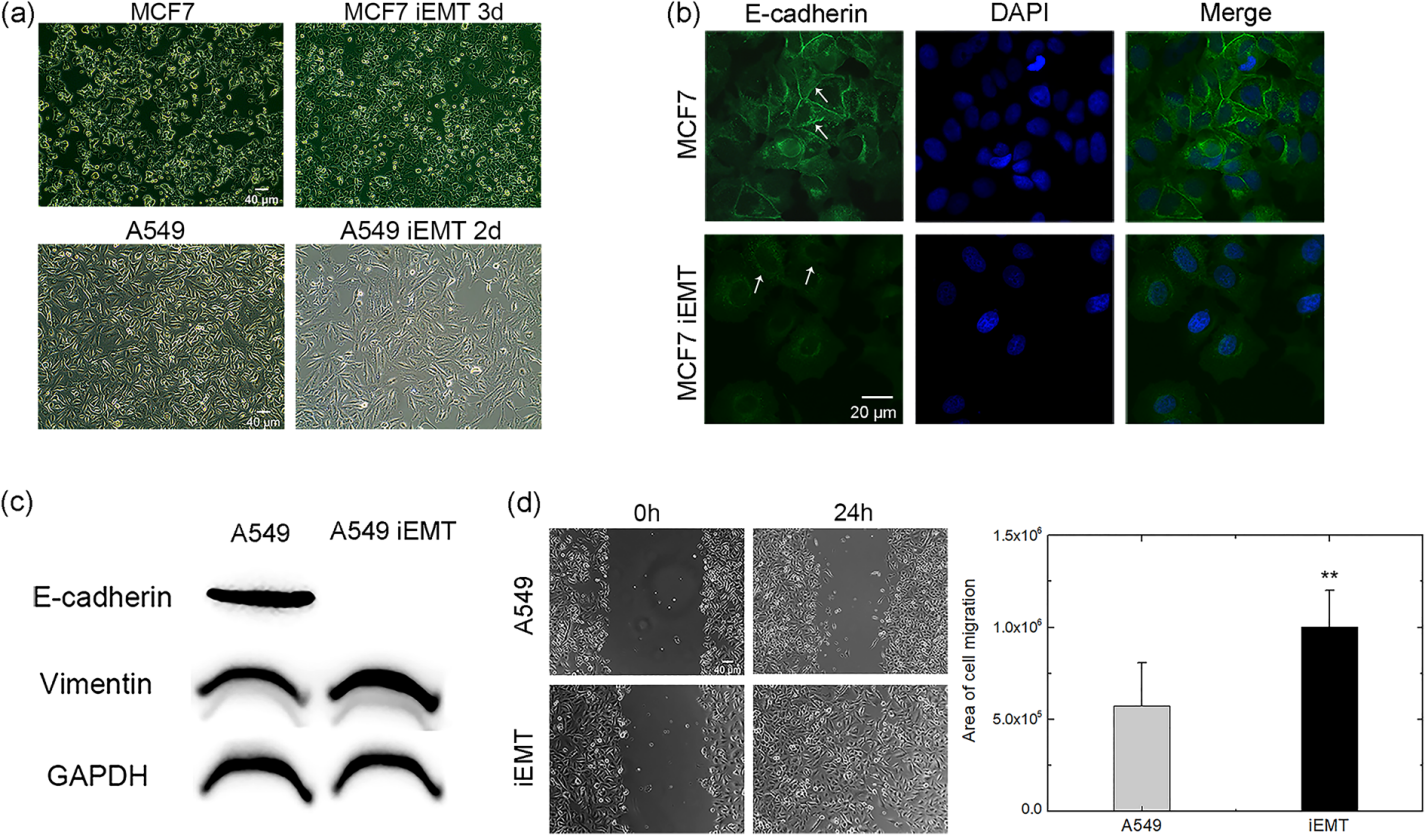
Characterization of EMT-induced cells. (a) Morphologies of MCF7 and A549 cells and the corresponding iEMT cells captured by a phase contrast microscope using a 10× objective lens. Scale bar = 40 *μ*m. (b) Fluorescence images of E-cadherin expression (green) in MCF7 cells and iEMT 5d cells. The white arrow shows the decrease in E-cadherin levels after EMT induction. Cell nuclei were stained with DAPI (blue). The merged image is a superposition of all the images. Scale bar = 20 *μ*m. (c) Expression of E-cadherin and vimentin protein in A549 cells and iEMT 2d cells, measured by western blot. (d) Migration ability of A549 cells and iEMT 2d cells as assessed by wound healing assay. The scratches were almost filled with cells 24 h after EMT induction. Scale bar = 40 *μ*m. The area of cell migration was analyzed by ImageJ. **P < 0.01.

To study the relationship between cell compressibility and EMT status, EMT was induced in cell lines derived from two cancer cell types (MCF7 and A549). The conditions for inducing EMT by growth factors are described in the Experimental section. The effectiveness of EMT induction was verified at the molecular and cellular levels using immunofluorescence, western blotting, and wound healing experiments. As expected, EMT induction transformed the cell morphology. The morphology of MCF7 cells changed from squamous to spindle-shaped, with a reduction in cell–cell contact [Fig. 4(a)]. The A549 cells transformed from a cobblestone epithelial morphology to a more fibroblast-like morphology [Fig. 4(a)]. Following EMT induction, biomarkers related to EMT showed corresponding alterations. In MCF7 cells, the epithelial biomarker E-cadherin was downregulated [indicated by the arrow in Fig. 4(b)], although the mesenchymal biomarker vimentin was not upregulated significantly (data not given). A similar phenomenon was apparent in A549 cells, where E-cadherin protein expression was significantly reduced, while vimentin protein expression was obviously increased [Fig. 4(c)]. A reduction in E-cadherin expression leads to decreased intercellular adhesion and increased cell motility.^19^ Vimentin has also been shown to promote cell motility.^24^ Indeed, in the wound healing experiment, the scratch was largely filled after EMT induction [Fig. 4(d)], indicating that EMT significantly increased the migration ability of cells.

**FIG. 5.**
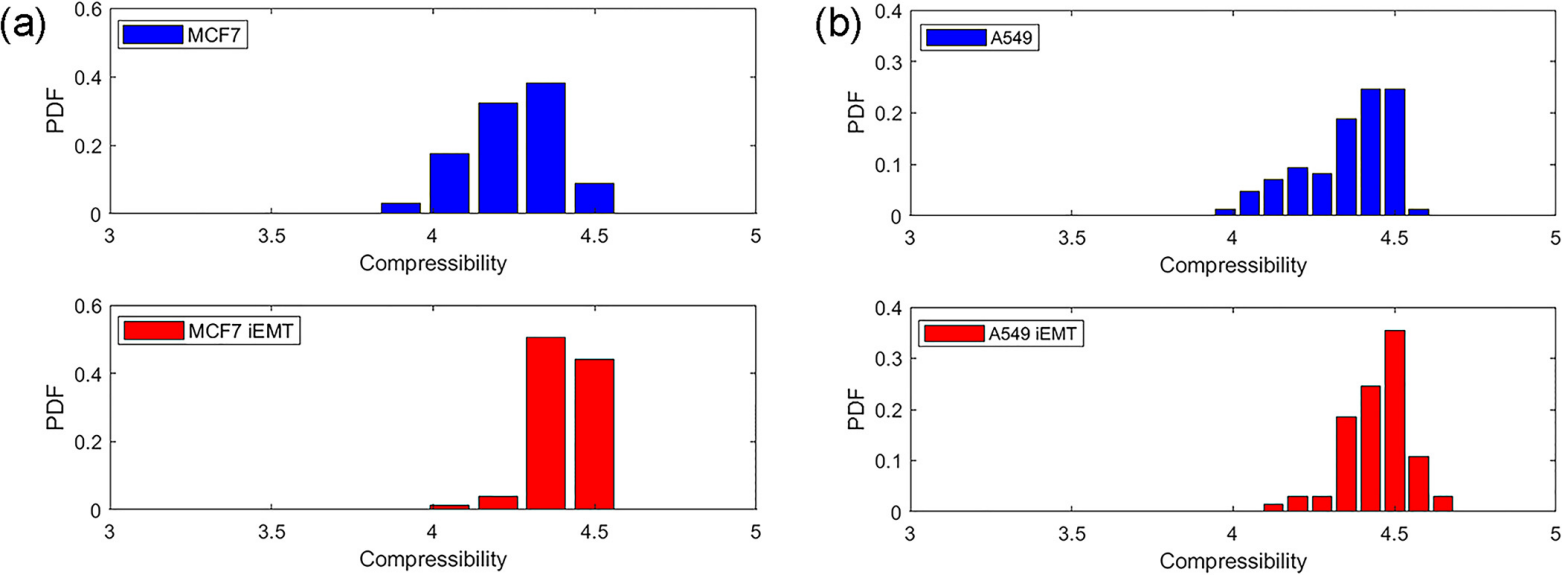
Probability distributions of cell compressibility in cells with or without EMT induction. (a) Probability distribution diagrams of cell compressibility in MCF7 cells (N = 34; blue column) and iEMT 3d cells (N = 77; red column). (b) Probability distribution diagrams of cell compressibility in A549 cells (N = 85; blue column) and iEMT 3d cells (N = 65; red column).

Further, the compressibilities of cells with or without EMT induction were compared using the acoustofluidic microdevice as described above. As shown in Fig. 5(a), the compressibility of MCF7 cells following EMT induction (4.41 ± 0.10 × 10^−10^ Pa^−1^) was significantly increased compared with that of MCF7 cells without EMT induction (4.24 ± 0.14 × 10^−10^ Pa^−1^, *P* < 0.01). Likewise, the compressibility of A549 cells was significantly increased from 4.35 ± 0.14 × 10^−10^ Pa^−1^ to 4.45 ± 0.10 × 10^−10^ Pa^−1^ (*P* < 0.01) after EMT induction [Fig. 5(b)]. These findings are summarized in Table I. Correlation analysis showed strong correlations between cell compressibility and metastatic potential indicators including migration and invasion. Cell compressibility and expression of vimentin (a biomarker of EMT) were also strongly correlated. Collectively, these results suggest that cell compressibility could be used to monitor the transition of cells to a highly invasive and aggressive state.

**TABLE I.** Pearson's Correlation analysis results. R is the Pearson's product-moment correlation coefficient.

A549	R	p-value
Cell compressibility/invasion	0.859	0.013
Cell compressibility/migration	0.923	0.003
Cell compressibility/EMT	0.844	0.017

Overall, the above results indicate that the changes in cell mechanics and migration characteristics after EMT enable cancer cells to migrate and enter the microvasculature, leading to distant metastasis.^25^ Studies have shown that the mechanical phenotype of cells is related to the cytoskeleton, including the cytoplasmic skeleton and nucleoskeleton. The organization,^26^ content,^27^ and content ratio^28^ of microfilaments and microtubules are important contributors to cytoplasmic skeleton. In addition, chromatin and nuclear lamins are determinants of the maintenance of nucleus mechanics and mechanotransduction of the nucleoskeleton.^29^ In particular, nuclear lamins have been shown to be associated with EMT-related pathways and metastasis.^30,31^ Therefore, our subsequent work will further investigate the relationship between cell compressibility and the cytoskeleton to elucidate the effects of cell mechanical phenotypes on cell migration and invasion.

### C. Limitations and future work

Cell mechanical properties are considered as promising unlabeled markers for the diagnosis of cancer metastasis. The acoustic flow microdevice in this paper provided a rapid and non-destructive method to measure cell mechanical properties (cell compressibility). It was worth noting that there are several important experimental parameters that could influence the measurements of cell compressibility. The influencing factors included not only cell parameters (e.g., cell diameter, density and acoustic energy density, etc.) but also flow parameters and the particle–wall interaction. In our measurements, the cell area was measured by the freehand selections option of ImageJ software. The maximum deviation of cell diameter measurements was ±2.5%, corresponding to a deviation in the measured cell compressibility of ±1.25%. Since standard beads are always introduced with the cells to determine the acoustic energy density, the measurement deviation of cell compressibility caused by the change of acoustic energy density will be small, which was reported less than 1%.^16^ The typical cell density spread of ±10 kg/m^3^ will cause a deviation in the measured cell compressibility of ±1.25%.^16^ Besides, the study of Garofalo *et al.* has made a very detailed discussion on the contribution of particle–wall interaction,^32^ which can be used as a reference for subsequent research. This suggests that these influencing factors need to be considered and carefully studied in order to improve the accuracy of measurement. In addition, based on the association between cell compressibility and EMT status that we have established, the further study will focus on the role of proteins related to cell mechanical properties (such as cytoskeleton) to explain the molecular mechanism of this association.

## IV. CONCLUSIONS

The compressibility of cells was measured using a specially designed acoustofluidic microdevice. The compressibility of cancer cells with different invasiveness and metastasis statuses was investigated, showing that the more invasive cells had higher compressibility. The correlation between cell compressibility and EMT status was further studied; the results showed that cell compressibility was significantly increased after the EMT process. In the view of the important role of EMT in cancer metastasis, cell compressibility has predictive diagnostic potential and could be used as a marker of malignant transformation and metastasis of cancer.

## Data Availability

The data that support the findings of this study are available from the corresponding authors upon reasonable request.
